# Systems biology perspectives on the carcinogenic potential of radiation

**DOI:** 10.1093/jrr/rrt211

**Published:** 2014-03

**Authors:** Mary Helen Barcellos-Hoff, Cassandra Adams, Allan Balmain, Sylvain V. Costes, Sandra Demaria, Irineu Illa-Bochaca, Jian Hua Mao, Haoxu Ouyang, Christopher Sebastiano, Jonathan Tang

**Affiliations:** 1 Department of Radiation Oncology, New York University School of Medicine, 566 First Avenue, New York, NY 10016, USA; 2 Helen Diller Family Comprehensive Cancer Center, University of California San Francisco, 1450 Third Street, San Francisco, CA 94158, USA; 3 Life Sciences Division, Lawrence Berkeley National Laboratory, 1 Cyclotron Road, MS977, Berkeley CA 94720, USA; 4 Department of Pathology, New York University School of Medicine, 566 First Avenue, New York, NY 10016, USA

**Keywords:** ionizing radiation, breast cancer, heavy ion radiation, modeling, initiation, promotion

## Abstract

This review focuses on recent experimental and modeling studies that attempt to define the physiological context in which high linear energy transfer (LET) radiation increases epithelial cancer risk and the efficiency with which it does so. Radiation carcinogenesis is a two-compartment problem: ionizing radiation can alter genomic sequence as a result of damage due to targeted effects (TE) from the interaction of energy and DNA; it can also alter phenotype and multicellular interactions that contribute to cancer by poorly understood non-targeted effects (NTE). Rather than being secondary to DNA damage and mutations that can initiate cancer, radiation NTE create the critical context in which to promote cancer. Systems biology modeling using comprehensive experimental data that integrates different levels of biological organization and time-scales is a means of identifying the key processes underlying the carcinogenic potential of high-LET radiation. We hypothesize that inflammation is a key process, and thus cancer susceptibility will depend on specific genetic predisposition to the type and duration of this response. Systems genetics using novel mouse models can be used to identify such determinants of susceptibility to cancer in radiation sensitive tissues following high-LET radiation. Improved understanding of radiation carcinogenesis achieved by defining the relative contribution of NTE carcinogenic effects and identifying the genetic determinants of the high-LET cancer susceptibility will help reduce uncertainties in radiation risk assessment.

## INTRODUCTION

Estimating the carcinogenic risks for different tissues in humans exposed to high-energy (HZE) densely ionizing radiation (IR) is difficult in the absence of exposed populations comparable with those exposed to sparsely IR from accidents, medical need and atomic bomb detonation. This issue is presently of significant concern for space travel. The United States National Aeronautics and Space Administration (NASA) defines the carcinogenic risks of radiation exposure as a type I risk that represents a demonstrated, serious problem with no countermeasure concepts that may be a potential ‘show-stopper’ for long duration spaceflight. The galactic cosmic radiation (GCR) environment is unlike any on earth because it includes all charged particle species from protons through to uranium at varying energies of up to tens of GeV/amu. Although 85% of the GCR consists of protons, high atomic mass (Z) and HZE particle radiation is of particular concern because the limited experimental data to date indicate that the relative biological effect (RBE) for carcinogenesis for densely ionizing HZE particles is several-to many-fold greater than sparsely IR.

Although protons are the most prevalent particle in GCR, the less abundant heavier ions (1%) are more effective biologically. During a 3-year flight in extramagnetospheric space, 3% of the cells of the human body would be traversed on average by one Fe ion [1]. The unique pattern of energy deposition incurred by HZE particle traversal is of primary interest for evaluating the biological effects of the GCR on astronauts [2, 3]. HZE particles have a high RBE for most biological endpoints [4]. However, some biological effects are not observed following sparsely IR [5], and some radiation effects, like genomic instability, do not show classic dose responses [6]. Hence, measurements of individual biological events do not necessarily describe the health consequence of radiation damage.

The challenge is to understand how cellular responses are integrated in a multicellular context resulting in health effects in humans. Ideally, estimates of cancer risk from human travel in space would be based on a mechanistic understanding of complex effects elicited by different types of radiation exposure. Radiation is a known carcinogen that has been implicated in the etiology of a number of human tumors, including breast cancer, lymphoma, liver carcinoma, sarcoma and glioma [7]. Radiation causes damage to DNA directly by the induction of double-strand breaks, or indirectly by generation of reactive oxygen species that damage sugar and base residues. The consequences of misrepaired lesions are mutations, translocations, deletions and amplifications, which are also hallmarks of cancer cells.

Effects that show linear or linear–quadratic dose responses, like mutations and cell kill, are attributed to energy deposition in the cell under study, i.e. targeted. However, in the last decade, the paradigm that is restricted to direct DNA damage after exposure to IR has been challenged by two classes of non-targeted effects (NTE): first, the demonstration that descendants of irradiated cells exhibit non-clonal damage (i.e. radiation-induced genomic instability) or altered phenotype; the second is the established evidence of so-called ‘bystander’ radiation effects, in which non-irradiated cells respond to signaling by irradiated cells [6]. NTE (e.g. media transfer) are functionally defined and occur by various mechanisms that include gap junctions, soluble factors and phenotype transition, which differ between cell types and between *in vitro* and *in vivo* models. The crucial questions are whether, under what conditions, and to what extent NTE contribute to human health risks following radiation exposure.

## CARCINOGENESIS IN CONTEXT

There is growing recognition that cancer as a disease results from a systemic failure in which many cells other than those with oncogenic genomes determine the frequency of clinical cancer. It has become increasingly evident that tissue structure, function and dysfunction are highly intertwined with the microenvironment during the development of cancer [8, 9] and that tissue biology and host physiology are subverted to drive malignant progression [10]. More than a quarter of a century ago, studies by Mintz and Pierce showed that malignancy could be suppressed by normal tissues [11, 12]. Many have even argued that disruption of the cell interactions and tissue architecture can even be primary drivers of carcinogenesis [13–17]. Recent experiments with engineered models have focused on identifying the type and means by which normal cells mediate the development of cancer [18–21].

Likewise, although the prevailing radiation health paradigm focuses on radiation-induced DNA damage leading to mutations, numerous studies over the last 50 years have provided evidence that even radiation carcinogenesis is more complex than generally appreciated (reviewed in [22]). Terzaghi-Howe demonstrated that the expression of dysplasia *in vivo* and neoplastic transformation in culture of irradiated tracheal epithelial cells is inversely correlated with the number of cells seeded [23–26] and identified TGFβ as a key mediator [27]. Barcellos-Hoff and Ravani used a p53 mutant mammary cell line to show that irradiating only the host increased the development of frank tumors [28]. Only 20% of transplants of unirradiated mammary epithelial cells developed small tumors in sham-irradiated mice, while 100% produced large aggressive tumors in mice irradiated with 4 Gy, 3 days before transplant.

If microenvironments induced by radiation can promote neoplastic progression in unirradiated epithelial cells, then events outside of the (targeted) box may significantly increase cancer risk. Understanding such non-targeted mechanisms can readily lead to testable hypotheses, and possible interventions, for health risks in future populations. We propose that radiation exposure culminates in cancer as a result of a combination of oncogenic mutations from targeted DNA damage together with selection due to NTE in irradiated tissues [22, 29]. Our overarching hypothesis is that cancer ‘emerges’ as a result of a complex, but ultimately predictable, interplay between TE and NTE in the context of host genetics and physiology [29]. Thus, TE like mutation must interact with NTE, but how and to what extent such interactions are affected by radiation quality is unknown.

## CANCER RISK AND NON-TARGETED EFFECTS

To evaluate whether NTE contribute to mammary carcinogenesis, we created a radiation chimera model in which the mammary glands of irradiated hosts were transplanted with oncogenically primed mammary cells. In the first set of experiments, a cell line, COMMA-1D, was injected into mammary fat pads from which the endogenous epithelium had been surgically removed [28]. This cell line gave rise to normal outgrowths in unirradiated hosts and rapidly produced tumors in mice that had previously been irradiated with 4 Gy. These data indicated that radiation could alter carcinogenic potential indirectly by modifying tissue interactions.

Recent radiation chimera experiments use *Trp53* null mammary tissue as the target epithelium [30, 31]. *Trp53* null tissue gives rise to grossly normal ductal outgrowths when assessed at 3 months post-transplantation. As shown by Medina and colleagues, who established this genetic chimera model, almost all *Trp53* null outgrowths generate a tumor over the course of 12–18 months [32–35]. The tumors are highly heterogeneous carcinomas that have many of the features found in human breast cancer, including differential expression of hormone receptors, and centrosome aberrations. More intriguingly, they also show diverse expression profiles that are similar to those used prognostically in human cancer [32–35].

Using *Trp53* null epithelium in the radiation chimera model provided significant new evidence of the importance of NTE [31]. First, fewer cancers develop within 1 year from *Trp53* null epithelium transplanted after mice have been irradiated with 400 cGy, which is attributed to reduction in hormone stimulation as a consequence of damage to the ovaries. However, consistent with the COMMA-1D experiments, tumors arose more rapidly in hosts irradiated with ≤ 100 cGy, and, once detected, tumors grew faster, even though the irradiation had occurred months before and the target epithelium had not been irradiated. More surprisingly, the frequency of estrogen receptor (ER)-positive tumors decreased from 60% to 40% in irradiated hosts. Our knowledge that ER-negative tumors in humans predominate in young (<45 years of age) women (and are more difficult to treat) suggests that host irradiation promotes more aggressive tumors. Notably, the risk of breast cancer in women treated with radiation for childhood malignancy is comparable with that of *BRCA1* mutation carriers, and these cancers are also more likely to be ER-negative than age-matched controls [36].

Expression profiling of *Trp53* tumors that arose in control vs irradiated hosts showed that the profiles were distinct, independent of ER status. Using bioinformatic molecular subtyping the *Trp53* null tumors were distributed between six clusters, but the distribution of tumor subtypes as a function of host irradiation was not significantly different. Yet host irradiation confers a distinct expression signature on tumor transcriptomes [31]. Since tumors arising in irradiated hosts were not enriched in a particular tumor subtype, the gene lists that define tumors arising in irradiated hosts can be considered metaprofiles that overlay intrinsic subtype. To test the relevance of this model to human cancer, the human homolog of genes that discriminated between tumors arising in irradiated vs non-irradiated mice was applied to radiation-preceded thyroid cancers and radiotherapy-associated sarcomas, both of which were segregated from sporadic cancers using the murine genes associated with tumors arising in irradiated hosts [37]. Using this approach on compiled datasets of profiles from sporadic breast cancers revealed significant clustering of ER-negative, basal-like breast cancers. The irradiated host gene list is highly enriched for genes associated with mammary stem cells (MaSC) and inflammation. Notably, both signatures are also evident in intact tissue shortly (1–4 weeks) after irradiation with 10 cGy. The functional significance of the stem cell signature was tested by analyzing stem cell function and markers in tissue from irradiated mice. The frequency of MaSC in tissue from adult mice exposed to 10–100 cGy during puberty was twice that of control mice. Based on the cell-of-origin hypothesis (reviewed in [38]) and the observation that tumors arising in irradiated mice were significantly more likely to be ER-negative, these data suggest that radiation exposure expands the pool of MaSC that, after neoplastic transformation, can give rise to ER-negative breast tumors [31].

All together these data support the hypothesis that NTE do contribute to radiation carcinogenesis and open new avenues of study of radiation effects on different processes that might be amenable to prevention strategies. IR is one of very few environmental exposures known to increase breast cancer risk [39], with the greatest risk conferred by exposure before the age of twenty [40]. Therefore, we examined the age dependence of our finding that expression profiles from tumors arising in irradiated mice and irradiated mammary glands are significantly enriched for a particular MaSC signature. This signature is accompanied by a demonstrable increase in mammary repopulating activity and increased Notch signaling in tissues shortly after radiation exposure and months prior to tumor development [31]. We set out to determine what mechanism affecting self-renewal is most likely operational in the irradiated mouse mammary gland. While Notch activation in the normal ductal luminal epithelium promotes MaSC, it can also mediate lineage commitment [41]. An alternative mechanism observed following high doses of radiation in bone marrow and intestine is stem cell loss, forcing self-renewal during tissue recovery (reviewed in [42, 43]). Notably, such radiation doses are 5–50 times greater than that used in radiation chimera mammary experiments (10–100 cGy). However, relatively low doses of radiation can also induce senescence [44], which might similarly affect self-renewal. We used radiation dose and quality effects to test the hypothesis that cell kill *per se* is a key event. Either increasing radiation dose or using Si-particle irradiation causes more cell kill, thus if repopulation following cell loss underlies the stem cell activity, we would expect a proportional effect on mammary repopulating activity. Yet both high dose and particle radiation increased MaSC signatures and repopulating activity similarly to that following low-dose exposure [30].

Another possibility is epithelial to mesenchymal transition (EMT), which is strongly associated with recapitulation of stem cell programs [45]. Exogenous TGFβ is a key signal for EMT [46, 47], and radiation induces TGFβ activation both *in vitro* and *in vivo*. Moreover, IR primes cultured human mammary epithelial cells to undergo TGFβ-mediated EMT [48, 49]. With the assistance of multiscale modeling (see below), we determined that radiation-induced signals, TGFβ and Notch, were key for the increased self-renewal that occurs in pubertal but not adult mammary glands [30]. Consistent with our cell-of-origin-based hypothesis, irradiating *Trp53* null outgrowths after morphogenesis is complete did not affect the distribution of ER-positive and -negative tumors. Connecting NTE and stem cell regulation to age may explain why radiation exposure at a young age confers the greatest breast cancer risk and provides a mechanism that may be amenable to intervention in susceptible populations.

## CANCER AND INFLAMMATION

Inflammation is the underlying response of the immune system to tissue damage. Paradoxically, the response necessary to restore homeostasis can become itself the cause of disease. Maladaptive inflammation is a main pathogenic mechanism driving chronic diseases, and is recognized as playing a key role in carcinogenesis [50]. The concept that inflammatory responses are necessary components of cancer development has recently been formalized by Mantovani *et al*. [51] in a two-pathway model: the intrinsic vs extrinsic. In the intrinsic pathway, genetic mutations lead to release by the transformed cells of pro-inflammatory factors recruiting innate immune cells. For example, oncogenic *ras* activates the transcription of the inflammatory cytokine interleukin-8 (IL-8). Other oncogenes such as *bcl-2* inhibit apoptosis, leading to necrotic tumor cell death and release of damage-associated molecular pattern molecules that activate innate immune cells via toll-like receptors [51, 52]. In both circumstances, the resulting host response is a smoldering inflammation that promotes tumor invasion and growth [51, 53]. In the extrinsic pathway, the chronic inflammation results from inability of the immune system to resolve an infection (e.g. hepatitis B) or from a deregulated immune response as in autoimmune diseases (e.g. inflammatory bowel disease). The persistent inflammation cooperates with pre-existing oncogenic mutations by providing the microenvironment that promotes cancer progression, but may also induce DNA damage resulting in new mutations [54, 55].

The innate immune system acts quickly to restrict injury and initiate wound repair and defense systems, depending on the nature of the damage. As a consequence, tumors contain a diverse inflammatory infiltrate. For example, macrophages functionally differentiated towards a phenotype characteristic of wound repair found in many solid malignancies play a central role in increased microvessel density and immunosuppression and are associated with reduced patient survival [56]. Importantly, recent data from Wright and colleagues explain earlier findings of radiation-induced genomic instability (GIN) in hematopoietic stem cells via inflammatory responses (reviewed in [6]). The most recent study shows that macrophages from irradiated mice can induce chromosomal instability in non-irradiated hematopoietic cells and that production of TNFα and reactive oxygen and nitrogen species by the macrophages were responsible for this effect [57]. Furthermore, Coates *et al*. showed that the mouse genotype affects macrophage phenotype, designated as M1 or M2, and that radiation exposure further amplifies the differential effect of genotype [58]. Together, these data support the hypothesis that cancer risk derived from exposure to radiation is the result of alterations in a network of cellular interactions, at the center of which is the innate immune system.

The contribution of radiation NTE to cancer incidence has just begun to be studied. Classic dietary intervention studies in experimental models suggest that it is a significant player. Burns and colleagues found that chronic exposure to dietary vitamin A acetate can prevent 90% of the malignant and benign neoplasias that occur in rat skin exposed to electron radiation and 50% of ^56^Fe ion beam-induced tumors [59]. Gene expression analysis suggested that ^56^Fe ion radiation significantly induced inflammation-related genes, including many in the categories of ‘immune response’, ‘response to stress’, ‘signal transduction’ and ‘response to biotic stress’, and that vitamin A reduced or blocked 80% of the gene expression alterations [60], consistent with the hypothesis that HZE NTE induce inflammatory processes that contribute to carcinogenesis.

How interplay between inflammatory cells and genetically mutated neoplastic cells promotes cancer development and progression remains a subject of intense investigation. Several important pathways have been identified. For example, IL-6 signaling plays a major role [61]. The main source of IL-6 is macrophages during acute inflammation, while T cells are the major source during chronic inflammation. Importantly, IL-6 orchestrates the transition from acute inflammation, dominated by granulocytes, to chronic inflammation, dominated by monocytes/macrophages and regulates, together with TGFβ, the differentiation of naïve T cells into the Th17 pro-inflammatory phenotype, thus influencing the type of adaptive immune response [62].

Cancer incidence in humans increases exponentially with age, with 75% of newly diagnosed cases occurring in susceptible populations aged 55 years or older. Given that space travelers will be adults for the foreseeable future, it is important to consider the basis for this relationship. Aging is associated with increased levels of chronic inflammation, which are thought to contribute to many age-associated diseases (including cancer), and increased serum levels of IL-6 have been reported in older individuals [63]. Interestingly, exposure to A-bomb radiation has also been associated with significant increases in serum IL-6 levels that are still detectable after many years [64]. These findings suggest the possibility that some of the radiation NTE that augment cancer risk in exposed individuals are, at least in part, mediated by induction of a pro-inflammatory environment similar to the aging process. If so, one might speculate that NTE and aging may synergize in terms of cancer risk.

An important implication of understanding the role played by the pro-inflammatory environment in radiation-induced carcinogenesis is the possibility of mitigating the consequences of radiation exposure with preventive use of anti-inflammatory agents. In fact, recent epidemiological data linking aspirin use to reduced risk of colorectal cancer development, a cancer type clearly linked to inflammation [65], suggest that such strategies could have an impact in reducing cancer risk in individuals exposed to radiation.

## MODELING RADIATION CARCINOGENESIS

Quantitative multistage carcinogenesis models have been proposed for identifying key mechanisms underlying radiation carcinogenesis and have been used to estimate radiation risk. A multistage theory of carcinogenesis was introduced very early [66, 67] to account for the observed power of age dependence in radiation-induced carcinomas. However, this model suggested five to seven rate-limiting stages, in contradiction with biological data. More recently, biologically based approaches addressed this contradiction by introducing the two-stage clonal expansion (TSCE) model where a cell leads to a tumor by two separate mutations and clonal expansion [68–70]. The TSCE model assumes that carcinogenesis occurs in four interdependent stages (initiation, promotion, transformation and progression). The first stage, ‘initiation’, is modeled with a constant mutation rate (µ_1_) and is typically thought to be caused by direct effects of chemical, physical or biological agents that irreversibly and heritably alter the cell genome, resulting in an enhanced growth potential. This potential is only realized, however, if the cell later undergoes ‘promotion’, the second stage of carcinogenesis. Promotion is often thought to be the rate-limiting step in carcinogenesis, since it has been shown that initiation alone is not sufficient to induce cancer [71]. Transformation is a stage that is modeled with a constant mutation rate (µ) in which a premalignant cell acquires an additional alteration and becomes a malignant cell. ‘Progression’ is the final stage leading from malignant cells to clinical cancer. Mutational models with integration of specific genetic mutations in tumor suppressor genes were originally introduced by Knudson [72]. The current paradigm of carcinogenic risk remains heavily focused on predicting mutations of the genome leading to silencing of tumor suppressor genes or to the activation of oncogenes.

However, TSCE does not include the considerable influence of intercellular and extracellular interactions in the tumor growth and predicts a final tumor that is unrealistic in that its cells are clonally identical. Tumors are in fact highly heterogeneous, and cell–cell and cell–extracellular matrix (ECM) interactions play a critical organizing role, and their impact on this expansion process should be included in future models. A tissue-based rather than cellular paradigm for carcinogenesis and tumor growth, which emphasizes the key role of cell–cell and cell–ECM interactions has gained considerable support [8]. For example, during angiogenesis a tumor manages to communicate with its microenvironment to elicit the proliferation of endothelial cells to form blood vessels that will supply the tumor with oxygen. Another illustration of this paradigm is the existence of cancer susceptibility genes whose mutations broadly affect genomic stability but are associated with cancer only in certain tissues (e.g. *BRCA1* for breast cancer, *APC* for colon cancer). This would suggest that the cellular and tissue context itself plays a role in causing the initiated cell to start proliferating.

Recent work introduced genomic instability into the TSCE model in order to fit colon cancer data better [73]. Fits were excellent but also suggested that radiation only played a small role in initiating genomic destabilization. The idea that non-mutational radiation effects play a critical role in destabilizing the genome is supported by the literature describing NTE on genomic instability [74–76]. Multicellular interactions typically lack mathematical formalism due to the difficulty of representing them as single entities such as cells. By modeling the irradiated tissue/organ/organism using systems biology approaches rather than a collection of non-interacting or minimally interacting cells, cancer can result as an emergent phenomenon of a perturbed system (29), which requires a new kind of formalism.

## AGENT-BASED RADIATION BIOLOGY MODELS

As pointed out by Pierce and Mendelsohn [77], what is important about a model is that it be useful (rather than complex), perhaps by providing new insights into data or a framework for further thought. Advances in computer science have engendered new approaches for modeling biological systems in ways that can formalize underlying assumptions about biology. Agent-based models (ABM) are a form of Monte Carlo models that naturally describe complex adaptive systems as the results of interactive components in various contexts [78]. ABM are non-deterministic codes originally developed for artificial intelligence. In a simulation, each agent behaves individually in response to its situation on the basis of a set of contextual rules. In the case of representing cells within a tissue, agents may execute various behaviors appropriate for the system, such as proliferation, differentiation or death. One key advantage of ABM is that it is easy to modify or add to existing codes, making them expandable to larger problems. Rather than changing a large number of equations or lines of code, as may be required in the case of a conventional mathematical model, a protein interaction can be introduced or modified simply by adding or changing a single rule that represents the interaction of interest. By avoiding the combinatorial explosion that would be necessary to model mathematically complex biological systems, agent- and rule-based representations hold promise for making modeling more powerful, more perspicuous, and useful to a wider audience in biology. Finally, because ABM allow easy expansion to larger scale systems, they may be a very useful tool for eventually predicting risk at the macroscopic level of a tissue or organism.

ABM have proven to be very useful in predicting emerging properties from complex systems [79–84]. Some studies have combined biological data measured by immunohistochemistry or biochemistry with behavior rules to create the representation of a tissue [85]. By applying such technology to model thymocyte development, Efroni and colleagues showed that competition between thymocytes for sites of stimulation could be important in generating the fine anatomy of the thymus [86]. ABM have been used to predict the long-term response of human tissue to IR. Enderling and colleagues used ABM to predict the responses of cancer stem cell populations to IR [87]. They showed that the three basic components of tumor growth (cell proliferation, migration and death) can have some unexpected effects on tumor progression and, thus, clinical cancer risk [88]. More specifically, increased proliferation capacities and limited cell migration in non-stem tumor cells lead to cell crowding, which inhibits tumor growth. In contrast, increasing the death rate of non-stem tumor cells leads to long-term tumor outgrowth by increasing the pool of cancer stem cells [89]. Stern and colleagues used previously validated ABM of epithelial *in vitro* wound response [90] to understand how IR can help the virulent bacterium *Pseudomonas aeruginosa* kill epithelial cells in irradiated intestines [90]. ABM of skin have recently also been introduced by von Neubeck and colleagues [91] to understand the effects of heavy ion radiation on tissue homeostasis.

Our own research focuses on radiation and breast cancer. We used ABM to examine how IR affects stasis [44], a senescence barrier observed in primary human epithelial cell cultures [92]. Unexpectedly, ABM revealed that competition can provide a proliferative advantage to a subpopulation resistant to stasis in primary cultures. A set of more elaborate ABM was then introduced and validated by accurately simulating the *in vitro* acinar morphogenesis of human mammary epithelial cells in 3D culture [93]. In order to evaluate the likelihood of different mechanisms leading to enrichment of stem cells in the irradiated mammary gland [31], we extended this set of model to simulate mammary gland morphogenesis and MaSC lineage commitment [30]*.* This extended set of ABM generated and monitored hundreds of millions of epithelial agents representing mammary stem, progenitor or differentiated cells in a 3D computerized matrix, representing an *in silico* ductal tree [30]. *In silico* predictions of the relative contribution of self-renewal, repopulation or senescence were then tested using experimental data from an MCF10A human cell line, in which basal and luminal cell populations were tracked in live and fixed specimens following exposure to IR. Together, the modeling and experiments indicated that the combination of cell proliferation during puberty with specific signals that increase stem cell self-renewal creates a window of opportunity for stem cells to expand. Our study provides a likely mechanism for the observation that women exposed to IR under the age of 20 have a greater risk of developing aggressive breast cancer than those exposed later in life [39]. Our results are also in good agreement with another computational model of increased cancer stem cells in irradiated tumors [94], although thought to be due to better DNA repair [95]. Hence, tumor cell survival is achieved by a shift from asymmetric to symmetric stem cell division during therapeutically fractionated radiation exposure [94].

The integration of a classic deterministic radiation-induced cancer model (such as the TSCE model of Moolgavkar and colleagues [69]) with stochastic models simulating complex tissue (including cell–ECM interactions, spatial organization and temporal dependence of growth factors, as described in this section) can lead to powerful predictive tools in radiation biology. Our group has already developed a more sophisticated multistage clonal expansion model that incorporates the impact of genomic instability on cancer progression [96]. Radiation-induced genomic instability and other NTE can increase the probability of transformation and enhance the malignant phenotype [6]; hybrid ABM–deterministic models may help us tease out the relative contributions of cancer initiation, promotion and progression in the context of irradiated tissue.

## SYSTEMS GENETICS

Many models of cancer risk and mitigation are focused on ‘targets’, i.e. the cell that will undergo neoplastic transformation or the genetic alterations that initiate and promote this event. We propose that targeted cancer initiation (defined as mutations resulting from misrepaired DNA damage caused by IR) is only half the story, and that non-targeted radiation-induced host biology is critical to the action of radiation as a carcinogen and in the development of clinical cancer. Unlike the random interaction of energy with DNA resulting in damage and mutation, tissue response to radiation is orchestrated and predictable, and may ultimately be amenable to intervention. If key signals that promote carcinogenesis in irradiated tissues are identified, then the irradiated microenvironment can be a therapeutic target for mitigating the long-term consequences of unavoidable radiation exposure during space travel.

A major component of cancer risk is heritable, i.e. polymorphisms passed through the germline can have in some cases a dramatic effect on the probability of developing cancer. We define ‘Systems Genetics’ as a process by which the effects of inherited polymorphisms on normal tissue architecture can be visualized, leading to the identification of critical components that can promote or prevent cancer development. Systems genetics approaches seek to integrate multidimensional datasets that encompass complex interactions between genetic polymorphisms, mRNA and protein expression, and disease phenotypes.

Gene expression levels can be influenced by complex interactions among cis- and trans-acting factors. One method for distinguishing these factors involves generating a genetically heterogeneous population (such as a mouse backcross population), measuring gene expression levels in normal tissue from multiple individuals in the population, and treating the expression level of each gene as a quantitative trait (expression QTL or eQTL) [97, 98]. Using this method, the cis- and trans- acting alleles influencing gene expression are decoupled from each other, and genes whose differential expression is due to cis-acting factors at a locus can be distinguished from genes under control of trans-acting factors at other loci. This allows us to create a network view of tissue architecture that comprises both structural and functional components of the tissue. Our previous studies applied this method to analysis of the mouse skin [98], but additional datasets on other tissues (including mammary gland, lung, and brain) are presently being generated. Since each network is generated from a population of ∼ 100 individual animals, we can use this approach to identify network components that are enriched in specific mice that are susceptible to cancer or one of its subphenotypes, such as inflammation. We can also investigate the effects of acute perturbation of the network by high- or low-LET radiation or by tumor development. By modeling the entire tissue as a system rather than a collection of cells, we gain a deeper understanding of how the perturbation of networks results in cancer.

We initially applied these systems genetics approaches to analysis of susceptibility to skin cancer induced by chemical DNA-damaging agents and tumor promoters [97–99]. A network view was created from gene expression profiles of skin from a population of interspecific backcross mice; within this population some animals were sensitive to carcinogen-induced tumor development and others were completely resistant. This enabled us to identify features of the normal skin architecture that are associated with tumor susceptibility or resistance. Our studies revealed that both cell-autonomous (cell cycle, stem cell lineage) and non-cell-autonomous (inflammation, innate immunity) components of the network were differentially expressed in the susceptible animals. Interestingly, the highly susceptible mice exhibited increased levels of anti-inflammatory genes within the inflammation-associated network, in spite of the observation that high inflammation is associated with tumor susceptibility [98, 100, 101]. Many genes related to the skin barrier function are located within the inflammation gene networks. By eQTL analysis the vitamin D receptor gene (*Vdr*) was identified as a master regulator of this network, with low levels of *Vdr* in backcross animals being associated with increased tumor susceptibility. Indeed, this connection is echoed in human populations with low levels of vitamin D in the serum being associated with increased cancer risk [102].

These genetic studies highlight the complex and sometimes opposing roles of inflammation in cancer development. Studies of mouse models have long established the important role of inflammatory agents in squamous cell carcinoma development [103]. The generally assumed route of skin carcinogenesis is from benign papilloma, to malignant squamous cell carcinoma (SCC), with some tumors undergoing EMT to progress to spindle cell carcinomas [104]. Interestingly, upon reduction of TPA-induced chronic inflammation fewer papillomas and squamous cell carcinomas (SCC) were observed, yet mice still developed aggressive spindle cell carcinomas [105]. This suggests that inflammation levels may result in a potential network rewiring, and these highly invasive tumors may arise from a different target cell population from papillomas and SCC [105]. Gene expression analysis of known inflammatory markers was also markedly distinct between SCC and spindle cell carcinomas. The distinct differences between the malignant SCC and highly invasive spindle cell tumors illustrate the need for two distinct therapeutic treatments. Anti-inflammatory drugs can have contradictory effects on skin tumor development [103, 106], and over-expression of pro-inflammatory cytokines such as IL-1 can prevent skin tumor formation in mouse models of chemically induced skin cancer [107]. In contrast, germline deletion of TNF-α, another potent pro-inflammatory cytokine, also confers resistance to skin tumor formation [108]. The role of inflammation in cancer is therefore highly complex, with possibly different consequences associated with acute vs chronic inflammatory conditions. This analysis underlines the necessity, and utility, of studying the system as a whole in order to understand how perturbation of networks results in cancer.

## SUMMARY AND CONCLUSIONS

Systems radiation biology seeks to integrate information across time and scale that are determined by experimentation. A key property of a system is that some phenomena emerge as a property of the system rather than of the individual parts. By modeling the irradiated tissue/organ/organism as a system rather than a collection of non-interacting or minimally interacting cells, cancer can be seen as an emergent phenomenon of a perturbed system [29, 97]. Our studies and that of others indicate that a biological model in which radiation risk is the sum of dynamic and interacting processes could provide the impetus to reassess assumptions about radiation health effects in a healthy astronaut population and spur new approaches to taking countermeasures. Given the current research evaluating the consequences of complex, multicellular radiation responses, broadening the scope of radiation studies to include systems biology concepts should benefit risk modeling.

## FUNDING

The preparation of this review article was supported by NASA Specialized Center for Research in Radiation Health Effects, NNX09AM52G.
